# Renal infarction in vascular Ehlers–Danlos syndrome masquerading as pyelonephritis

**DOI:** 10.1002/ccr3.1639

**Published:** 2018-06-13

**Authors:** Khalid M. Dousa, Kashif Khan, Ben Alencherry, Lin Deng, Robert A. Salata

**Affiliations:** ^1^ University Hospitals Cleveland Medical Center Case Western Reserve University Cleveland OH USA; ^2^ Division of Infectious Diseases and HIV Medicine University Hospitals Cleveland Medical Center Cleveland OH USA

**Keywords:** pyelonephritis, renal infarction, vascular Ehlers–Danlos syndrome

## Abstract

Symptoms associated with numerous diseases can be indistinguishable from those of the urinary system disorders because receptors of many visceral organs as well as the body wall transmit sensation through pain fibers shared with the kidneys. Disregarding important family background of genetic disorder can be detrimental for some patients.

## 1 CASE PRESENTATION

**Figure 1:**
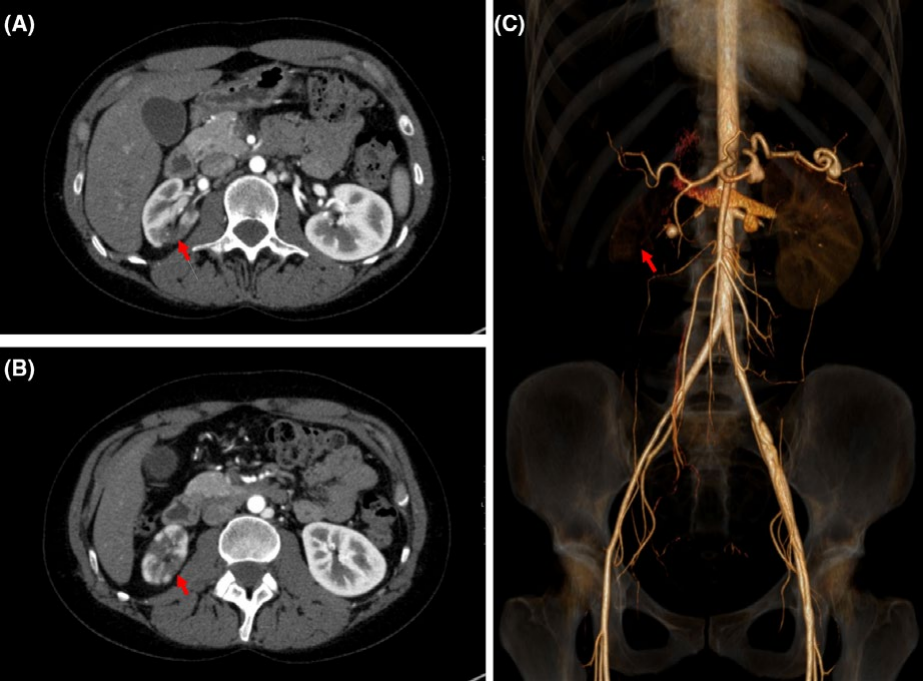
Computed tomography angiogram (CTA) scan showing diffuse cortically based, hypodense areas with nearly no perfusion of the renal parenchyma in the posterior aspect of the upper and lower pole (Panel A and B); 3‐dimensional reconstruction of the CTA demonstrated similar findings (Panel C)

A 29‐year‐old female presented to the emergency department with a 1‐day history of acute right flank pain, fever, and vomiting. She had a known history of a mutation in *COL3A1* gene associated with vascular Ehlers–Danlos syndrome (vEDS) and a strong family history of arterial aneurysms and rupture. On examination, her abdomen was soft and not distended, and with costovertebral angle tenderness. White blood cell count was elevated at 15 000 cells per cubic millimeter (normal range, 4000‐11 000). Urinalysis showed 5‐20 white blood cells per field with small leukocyte esterase. An abdominal computed tomography (CT) without contrast showed multiple hypo‐densities at the right kidney, initially interpreted as “severe pyelonephritis” but ultimately thought to be related to multiple renal infarcts. Because of the clinical suspicion of kidney infarction, CT angiogram was pursued and showed asymmetric contrast enhancement of the right kidney with nearly no perfusion of the renal parenchyma in the posterior aspect of the upper and lower pole (Figure 1, Panels A and B). A 3‐dimensional reconstruction of the CT demonstrated similar findings (Figure 1, Panel C). Renal duplex had findings consistent with renal artery dissection. The patient was admitted to the hospital, where she received supportive care and heparin therapy and beta‐adrenoceptor blocker. Antibiotics were withheld, and both blood and urine cultures were without growth. Over the next 3 days, her abdominal symptoms resolved.

## 2 DISCUSSION

Ehlers–Danlos syndrome (EDS) refers to a group of connective tissue disorders characterized by joint hypermobility and tissue fragility. Of the 13 described forms of EDS,^1^ vascular EDS (vEDS, also known as type IV EDS) is inherited in an autosomal dominant fashion and is the rarest and most severe form due to mutation of the *COL3A1* gene encoding type III collagen.^2^ Individuals with vEDS tend to have decreased amount of type III collagen and are at an increased risk of having spontaneous arterial, bowel, or uterine rupture.^3^ Although the prevalence of vEDS is not well studied, it accounts only for about 4 percent of all EDS cases.^4^

Our patient’s personal and family history were suggestive of the milder forms of vEDS, and she had no musculoskeletal signs or symptoms. She was diagnosed by genetic testing guided by her family history of arterial dissection, hollow viscus rupture, and sudden death among first‐degree relatives. Her father was diagnosed with ruptured aneurysm/dissection of small abdominal artery at age 30. She has a paternal uncle diagnosed with Ehler–Danlos syndrome in his second decades of life following splenic rupture from a trauma sustained in motor vehicle accident and found to have a COL3A1 mutation associated with vEDS. Her paternal grandfather was diagnosed with popliteal and superficial femoral artery aneurysms and died at the age of 80. Her paternal grandmother died during childbirth due to uterine rupture. The specific COL3A1 mutation in her family is likely to result in a lack of protein expression from the disease allele or in a protein chain that is incapable of incorporating into type 3 collagen fibrils. While vEDS has varying severity, routine complaints such as abdominal pain must be thoroughly evaluated in these patients for rare, catastrophic manifestations, including fatal hemorrhage.^5^

Renal infarction is an uncommon disease, with an estimated incidence rate of about 0.004%‐0.007% of ED visits.^6^ Due to its nonspecific presentation, it is frequently mistaken for other common diseases such as nephrolithiasis, pyelonephritis, lumbago, or other abdominal lesions delaying the diagnosis on average by 3‐5 days.^7^ Undiagnosed renal infarction poses risks of persistent symptoms, development of acute and chronic renal dysfunction, and an overall increased risk of mortality.^8^ Our patient’s clinical presentation mimicked pyelonephritis, a very commonly encountered presentation in young females. Adequate understanding of the vEDS disease process including its vascular manifestations such as renal infarction and renal artery dissection led to a prompt diagnosis in our patient even in the setting of misinterpreted initial cross‐sectional imaging and earlier treatment preventing potential complications.

Currently, the only intervention that has proven effective in reducing vascular complications in patients with vEDS is administration of beta‐adrenoreceptor antagonists, and this is based on the only multicenter randomized trial conducted by Ong et al (2010). During a median follow‐up period of 47 months, incidence of arterial complications was reduced by threefold. vEDS complications are life‐threatening because of weak arteries, bowel, or uterus, which can lead to spontaneous ruptures. Germain and Herrera‐Guzman^10^, ^11^ explained that invasive procedures are contraindicated due to risk of rupture and bleeding. Our patient did not require surgical intervention and had cessation of abdominal pain with no subsequent renal impairment or further clinically apparent vascular insults. She was discharged on treatment consisting of anticoagulation and beta‐adrenoreceptor antagonist Nadolol. At 3‐month follow‐up, her abdominal pain resolved, renal function remains stable, and no further vascular accidents occurred.

In addition to medications, a multidisciplinary team approach for management of patients with collagen vascular disease is crucial. Interventions in the form of avoiding contact sports, medical alert bracelet with the diagnosis labeled, avoidance of medications that elevates blood pressure, psychological treatment, prenatal diagnosis, and counseling about pregnancy complications and risks have been suggested by.^12^

In summary, a heightened clinician awareness is needed for major vascular complications in patients with vEDS, including arterial dissection, aneurysm expansion, and rupture. To date, the commonly pursued interventions are beta‐blocker therapy and avoidance at all costs of endovascular or open repair due to vessel fragility and risk of iatrogenic complications. Ongoing research is needed for targeted gene therapy and improved screening of the disease.

## CONFLICT OF INTEREST

None declared.

## AUTHORSHIP

All authors participated in drafting the article and revising it critically for intellectual content. KMD and RAS: analyzed and interpreted the laboratory findings. KMD, KK, BA, and DL: drafted the manuscript. KMD, DL, and RAS: made a critical review of the manuscript.
